# Extracts from the Records of the Atlanta Medical Society

**Published:** 1867-03

**Authors:** W. S. Armstrong

**Affiliations:** Demonstrator of Anatomy, Atlanta Medical College


### ARTICLE II.

*Extracts from the records of the Atlanta Medical Society.* By W. S. Armstrong, M. D., Demonstrator of Anatomy, Atlanta Medical College.

Dr. H. S. Orme reported the progress of a case of Tape Worm, mentioned at a former meeting. Under the use of Koosso, the patient discharged about twelve feet of the worm, and has not since felt any unpleasant symptoms. The remedy was given in infusion, in the proportion of half an ounce to twelve ounces of water—all to be taken at once.

Dr. R. C. Word reported a case of protracted hemorrhage from a leech bite, on the neck of a child, which he finally succeeded in arresting by the application to the bleeding point of pledgets of lint, saturated with collodion, and secured by strips of adhesive plaster.

Dr. D. C. O'Keefe reported a case of paralysis of the bladder in a lying-in woman, which commenced some days before labor came on, and lasted two or three weeks after its termination. The patient, at the time of her confinement, was extremely feeble from the existence of diarrhœa and deranged digestion for the last four or five months of gestation. The labor was easy and natural, and terminated in a few hours. The diarrhœa continued for nearly two months after delivery, and the condition of the patient was regarded hopeless for several days. The use of the catheter was always required to relieve the bladder, for the period above mentioned. This want of power in the bladder is of no uncommon occurrence following labor, but usually passes off in a few days. The explanation of it is found in the pressure of the child's head upon the hypogastric and sacral fluxus of nerves; and the temporary constipation of the bowels following labor may be accounted for in the same manner. The delay in the restoration of the contractile power of the bladder in the present case, was due to the great debility of the patient.

The treatment upon which she recovered consisted mainly of the liberal use of brandy, and a combination of Ext. Hyos. and Sulph. Morph.

Dr. D. C. O'Keefe desired to call the attention of the Society to the unusual prevalence of Malarial Fever in this city and vicinity during the present year, October, 1866; and in attending to cases of the kind mentioned, the occurrence of a case of malignant intermittent, or congestive fever, which proved fatal. This case had been one of simple quotidian intermittent fever for ten days, with a daily morning paroxysm. On about the tenth day he had a congestive chill, from which he never reacted. During this time he had been under the treatment of a Homœopathic practitioner, and Dr. O'Keefe saw him for the first time in a sinking condition, two hours before his death.

In a particular neighborhood of the southern portion of the city, he found most of the cases he had been called upon to treat. He considered that the three conditions necessary for the production of the disease to be heat, moisture, and the decomposition of vegetable matter.

Dr. J. G. Westmoreland had noticed in the same neighborhood alluded to by Dr. OKeefe, the prevalence of intermittent fever, and to such an extent as to attact whole families. In one instance every member of the family had the disease, and when relieved, suffered from frequent relapses. The cause to which the malaria in this neighborhood is attributed, is the partial draining of a fish pond.

Dr. W. F. Westmoreland desired to call attention to a particular character of fever prevailing to some extent in the city, exhibiting symptoms of ordinary remittent fever, containing from ten to twenty-one days unamenable to the free use of quinine.

Dr. Alexander thought that the type of fever referred to was observed in 1852, in this locality, at the commencement of the epidemic of typhoid fever, which prevailed that year. His opinion was, that such cases are not sbject to arrest by quinine, but are more profitably treated as ordinary typhoid fever.

Dr. J. M. Johnston had under treatment a case of fever answering to the description of those alluded to, in which the supporting and expectant plan of treatment has been adopted with the prospect of success.

Dr. J. M. Johnston also mentioned a case of brake-bone fever, suffering from excessive pain in the limbs. The case in question was recently from Columbus, Georgia, where this character of fever is prevailing as an epidemic.

Dr. J. G. Westmoreland desired the opinion of the members in regard to the fatal character of the disease as reported in the newspapers.

Dr. Knott said he had a limited acquaintance with Dengue, only in his own person, which terminated in three or four days without treatment.

Dr. Alexander had seen several, which did not yield to quinine, but terminated favorably in a few days—leaving a disposition to jaundice.

After the foregoing remarks, Dr. J. G. Westmoreland read a private letter, received a few days before, from Dr. Bacon, of Columbus, Georgia, giving a practical statement of the probable cause, character, symptoms, and treatment found most successful in this disease as it prevailed in that city the present season.

He writes, we attribute the disease to a peculiar poisonous atmosphere, caused by the exposure of the deposits on the bottom of the river (which had been covered with water for the last twenty years) to the action of the sun. The water was drawn off in July. The first case of brake-bone fever occured on the 6th day of September.

The disease attacks every one suddenly. The first symptoms are pain and soreness on pressure of the eyes, and pain in the head, sometimes excruciating, with pain in the back, and aching in the back part of the pelvis.

In some cases this is all the pain that is suffered.

In others, in addition to the above, every joint and bone and muscle aches; and when you ask them where hurts, they will tell you they hurt all over. I heard a little boy who was suffering very much, tell his father both of his thighs were broken. Fever accompanies these symptoms—sometimes of a high grade, though generally the reverse of this. The pulse and skin frequently are very little above the natural standard. During this first paroxysm, which lasts from six or eight to thirty-six or forty-eight hours, according to the treatment used, there is frequently vomiting of the contents of the stomach, followed by vomiting of bile. When the patient goes to bed, he wants blankets piled on him, and complains, whenever he moves, that cold streaks run over him. This constitutes the first paroxysm of this painful and disagreeable disease, which, with proper treatment, is almost always the last; though there is occasionally a case, where a light attack of fever supervenes on the third or fourth day.

There is an eruption on the skin in almost every case, with troublesome itching.

No matter how mild or severe the attack may have been, the patient is always left in a weak and feeble condition.

The mouth tastes horribly. The tongue is generally coated with a cream-like coating, that scarcely ever cleans off as long as the week, mean feeling stage last, which is generally from a week to ten days after they commence walking about. During the greater part of this period, there is a perfect loathing of all kinds of food. * * * * *

In fact, all who had brake-bone fever, say that for a week after they get about, they feel worse than at any time before in the whole course of their lives. Language, they say, fails to describe their feelings.

There is more or less nausea in every case. Persons who are rheumatic, or have a syphilitic taint, suffer for some time with pain in the various joints. My idea of the disease is, that it is situated in the nervous system, including, of course, its great center—the brain. I do not look upon it as inflammation, but a peculiar morbific effect, the result of a specific poison existing in the atmosphere.

I forgot to mention above, that in the large majority of the cases that have occured in females who menstruate, this disease has brought on the discharge again, though they may have passed through their regular period only a week or ten days previously. I attended one lady who had an infant only five weeks old. The discharge came on, and went regularly through as it did in others. Dr. Terry told me that he had two patients, one had seen nothing for three and the other five months. During an attack of brake-bone fever, both had a return of their perriod.

The treatment of this disease is very simple. When called to a case, if I find that the patient has not taken something to act on the bowels, I generally give a mercurial purge, if the pains are not very severe; but if they are, I give a full dose of epsom salts in pepper tea. I use the pepper tea on account of chilliness complained of, though sometimes, when the stomach is very irritable, I give simply a seidlitz powder every hour until the bowels are moved. My object is to have the bowels acted on as soon as possible, so that the system will be prepared for the introduction of opium, which is the great remedy in this disease. As soon as two or three actions have been produced, I give a pill composed of opium and camphor, one grain each. This is repeated in two hours, and then again every four hours until four or six pills are taken.

If, on the second or third day, fever returns, a seidlitz powder is ordered every two hours until an action is secured. This, however, seldom occurs when opium has been used. Quinine is hurtful in this disease.

It increases the nausea and general distress.

Large mustard plasters used at any time, but particularly in the commencement of the attack, afford great relief and comfort; also, hot foot baths and hot applications to the feet. Let the patient drink at all times as much hot or cold lemonade (*without ice*) as he desires.

After this I tell the patient, if he feels pain any where, to take two spoonsful of parragoric every hour, until relieved; also, to eat and drink whatever he wishes, and to go where he desires, feeling sure, from personal experience, that they have no disposition to commit excesses in either.

I have not yet seen or heard of a relapse from this disease, nor a return of it in the same person during the same season.

I have not lost a case of it, nor have I heard of a death from this cause, unless some old persons who died here this fall had it.

The disease, in my opinion, is not contagious, but caused by a peculiar poison in the atmosphere. I hope that this desultory communication may prove satisfactory.

